# Epigenetic Regulation of B Lymphocyte Differentiation, Transdifferentiation, and Reprogramming

**DOI:** 10.1155/2012/564381

**Published:** 2012-08-26

**Authors:** Bruna Barneda-Zahonero, Lidia Roman-Gonzalez, Olga Collazo, Tokameh Mahmoudi, Maribel Parra

**Affiliations:** ^1^Cellular Differentiation Group, Cancer Epigenetics and Biology Program (PEBC), Bellvitge Biomedical Research Institute (IDIBELL), Avenida Gran Via s/n km 2.7, 08907 L'Hospitalet de Llobregat, Barcelona, Spain; ^2^Department of Biochemistry, Erasmus University Medical Center, Ee622, P.O.Box 2040, 3000 CA Rotterdam, The Netherlands

## Abstract

B cell development is a multistep process that is tightly regulated at the transcriptional level. In recent years, investigators have shed light on the transcription factor networks involved in all the differentiation steps comprising B lymphopoiesis. The interplay between transcription factors and the epigenetic machinery involved in establishing the correct genomic landscape characteristic of each cellular state is beginning to be dissected. The participation of “epigenetic regulator-transcription factor” complexes is also crucial for directing cells during reprogramming into pluripotency or lineage conversion. In this context, greater knowledge of epigenetic regulation during B cell development, transdifferentiation, and reprogramming will enable us to understand better how epigenetics can control cell lineage commitment and identity. Herein, we review the current knowledge about the epigenetic events that contribute to B cell development and reprogramming.

## 1. Introduction

**Figure 1:**
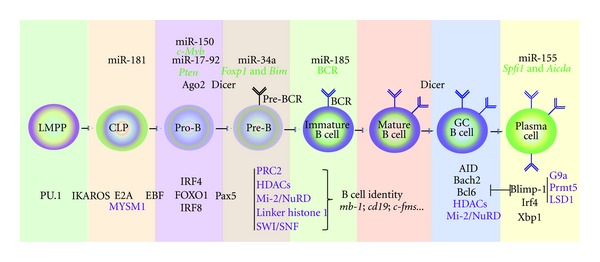
Scheme for B cell development. Successive stages of B cell differentiation and the key transcription factors and epigenetic regulators involved are shown. The epigenetic regulators that cooperate with specific transcription factors at every cell differentiation step are in purple. MicroRNA transcript targets are in green.

Hematopoietic stem cells (HSCs) give rise to mature B cells through the sequential differentiation of lymphoid progenitor cells. Long-term HSCs (LT-HSCs) have the ability to self-renew and reconstitute the entire immune system by differentiating into short-term HSCs (ST-HSCs). ST-HSCs differentiate into multipotent progenitors (MPPs) that then branch into common myeloid progenitors (CMPs) and lymphoid-primed multipotent progenitors (LMPPs). CMPs further differentiate into erythrocytes and megakaryocytes, whereas LMPPs retain the capability to give rise to myelomonocytic or lymphoid lineages [1, 2]. LMPPs become common lymphoid progenitors (CLPs) [3], which have the potential to differentiate into B and T lymphocytes as well as natural killer (NK) cells [4, 5]. Once committed to the lymphoid lineage, further differentiation steps lead to the formation of pro-B and pre-B cells, which are the early B cell precursors for immature B cells, the terminally differentiated plasma cells and germinal-center B cells (Figure 1).

Every step in B cell development is characterized by the activation of the specific genetic program characteristic of the new intermediate/progenitor generated and the repression/extinction of the genetic program of the previous cellular state. To achieve this, the different differentiation steps are tightly regulated at the transcriptional level. In recent years, the theory of the existence of networks of lineage-specific and identity-transcription factors responsible for establishing particular genomic landscapes has gained credence [6]. In the case of lymphocyte development, the transcription factors Ikaros and PU.1 are critical for the cellular commitment of LMPPs to the lymphoid lineage [2]. Subsequently, early B cell specification depends on the action of E2A, EBF, and FOXO1, whereas Pax5 is required for proper B cell development and for maintaining B cell identity [7–12]. Finally, during later developmental stages, the transcriptional repressors Bcl6 and Blimp-1 are crucial for the generation of germinal-center B cells and plasma cells, respectively [13–17] (Figure 1).

The picture of the hierarchical network of transcription factors that mediate the epigenetic signature needed to regulate the specific transcriptome of the fate of B-cells during their development has begun to emerge [18–20]. For example, Pax5, whose expression is induced by E2A and EBF, recruits chromatin-remodeling, histone-modifying and transcription-factor complexes to its target genes to activate the transcription of B cell-specific genes, and to silence lineage-inappropriate genes [19]. Extensive efforts have been made to elucidate the epigenetic mechanisms underlying the gene rearrangements of various components of the B cell receptor (BCR) [21–23]. Thus, epigenetic regulation is a critical event in B lymphocyte development. The relevance of transcription factors to the establishment and maintenance of cell-lineage identity has also been demonstrated in cellular reprogramming experiments [24–27]. The epigenetic mechanisms involved in the reprogramming and transdifferentiation of B cells have also been a focus of study in recent years.

Nucleosomes are the basic unit of the chromatin. They comprise 147 bp of DNA wrapped around a histone core, which contains two copies each of H2A, H2B, H3, and H4. This core is important for establishing interactions between nucleosomes and within the nucleosome itself [28]. Depending on the epigenetic modifications on the histone tails and in the DNA, chromatin can adopt different structural conformations that are correlated with its active, permissive (primed), or repressive status. The four main mechanisms by which epigenetic regulation occurs are DNA methylation, histone modification, chromatin remodeling, and regulation of gene expression by the action of noncoding RNAs. The methylation of cytosine residues at CpG dinucleotides (methyl-CpG), which is generally associated with transcriptional repression, is accomplished via the action of DNA methyltransferases (DNMTs) [29]. Methyl-CpG-mediated transcriptional repression can be explained by two nonmutually exclusive molecular mechanisms. First, methylation of DNA can interfere with the accessibility and recruitment of transcription factors to their DNA-binding sites. Second, DNA methylation results in the recruitment of methyl-CpG-binding proteins (MeCPs and MBDs) in association with corepressor complexes. Both mechanisms lead to the transcriptional silencing of the methylated genes [29]. The posttranslational modification of histones is another important epigenetic regulatory mechanism. Histones can be posttranslationally modified by a variety of enzymatic modifications, including acetylation, methylation, phosphorylation, sumoylation, and ubiquitination among others [30]. While acetylation is generally considered to be a mark of transcriptional activation, histone methylation can result in either transcriptional activation or repression, depending on the residue that is modified. In this regard, acetylation of histone H3 on lysine 9, 14, and or 18 (H3K9ac, H3K14ac, H3K18ac) is associated with transcriptional activation and considered “histone active marks.” In the case of histone methylation, di- and tri-methylation of histone H3 at lysine 4 (H3K4me2, H3K4me3) are associated with transcriptional activation and therefore considered an active mark, whereas trimethylation of H3 at lysine 27 (H3K27me3) is found to be enriched at silenced genes and considered to be a repressive histone mark [28, 30]. Trimethylation of histone H3 at lysine 9 (H3K9me3) has also been characterized as a mark of transcriptional repression [30]. However, different reports suggest that it can also represent transcriptional activity [31, 32]. Another mechanism of epigenetic regulation involves the action of chromatin remodelers, which are multi-subunit complexes that use the energy from ATP hydrolysis to change the location or conformation of nucleosomes, resulting in increased or decreased DNA accessibility [28]. Chromatin-remodeling complexes can be divided into four groups, characterized by core ATPase subunits. Based on the defining ATPase, they are referred to as the SWI/SNF, ISWI, CHD, and INO80 families of remodelers [28]. Finally, microRNAs (miRNAs), a type of small noncoding RNAs, have been shown to anneal to 3′UTR of cognate mRNAs, leading to mRNA instability and/or the inhibition of translation, thereby making it possible to modulate the proteome of the cell [29].

In this papre we will summarize the recent advances in our understanding of the epigenetic mechanisms controlling B cell development and reprogramming.

## 2. B Cell Development: Early Specification towards the Lymphoid Lineage

When cells are at the LMPP stage, two transcription factors, Ikaros and PU.1, play critical roles in the early cellular specification towards the lymphoid lineage. Mice homozygous for a germline mutation in the Ikaros DNA-binding domain present a block at early lymphocyte development and therefore lack lymphocyte progenitors, T and B lymphocytes, as well as natural killer cells [33, 34]. More recently, Ikaros was shown to be a crucial transcription factor for the commitment of LMPPs into CLPs, clearly demonstrating its key role in the early cellular decision to undergo lymphocyte development [2]. LMPPs derived from Ikaros-null mice lack B cell potential and do not express *Flt3*, *Il-7r*, *Rag1* and *Rag2*, which are important genes for lymphoid commitment [2]. Mechanistically, Ikaros can either activate or repress transcription of target genes, depending on the recruitment of coactivators or corepressors. For example, in T cells, Ikaros has been shown to recruit corepressor or chromatin remodeling complexes in order to either repress or activate specific targets [35–37]. However, how Ikaros mediates the epigenetic regulation of its target genes during the differentiation of LMPPs into CLPs remains to be elucidated.

Likewise, the transcription factor PU.1 is crucial for the commitment of LMPPs to the lymphoid lineage. Strikingly, PU.1 is also required for the generation of GMPs and macrophages. In fact, mice deficient for PU.1 die around birth and lack B, T, NK and myelomonocytic cells [38, 39]. The promiscuity of PU.1 in regulating gene expression in different cell types raised the general question of what the mechanism of action is of a given transcription factor in different cell types. In this regard, Heinz et al. recently identified the genomewide binding sites of PU.1 in splenic B cells, macrophages and B cell progenitors [40]. They found that PU.1 cooperates with cell-type-specific transcription factors to activate the cisregulatory elements required for the development of a particular cell type. For example, in CLPs and pro-B cells, E2A induces PU.1 binding at B cell-specific genomic sites that contain closely located PU.1 and E2A binding motifs [40]. In addition, PU.1 binding initiates nucleosome remodeling, followed by H3K4me enrichment at many specific genomic regions [40]. These data could lead us to speculate that cooperation between PU.1 and Ikaros might be crucial for the activation of specific genes required to specify LMPP into CLPs. Also, the identity of Ikaros and PU.1 epigenetic partners remains unknown. This matter awaits investigation.

## 3. B Cell Development: Early B Cell Commitment

B cell development is characterized by the generation of the BCR, which consists of a heavy and a light immunoglobulin chain, IgH and IgL, respectively. The expression of the BCR subunits *VpreB*, *λ5*, and *mb-1* (*Cd79a*), and the initiation of D-J rearrangements at the IgH locus defines early B cell commitment [41]. The specification of CLPs in the B cell lineage requires two transcription factors, E2A and EBF1, which have been shown to activate the expression of genes essential for the formation of pro-B cells [42]. E2A and EBF knockout mouse models are phenotypically similar, and both transcription factors are considered to play key roles in initiating B lymphopoiesis. E2A-deficient mice show arrested B cell development at the pre-pro-B cell stage with compromised D-J rearrangements at the IgH locus and a lack of expression of *Rag1*, *mb-1*, *I*ν**, *λ5*, *Cd19*, and *Pax5* genes [7–9]. More recently, it was shown that conditional deletion of E2A in pre-B cells did not result in a complete loss of expression of its target genes, indicating the involvement of E2A in the early steps of B cell commitment [43]. Similar to E2A, EBF is also known to play a crucial role in initiating B cell development. Mice lacking EBF do not express *Rag1*, *Rag2*, *mb-1*, *B29* (*Ig*β**), *λ5*, *VpreB*, *cd19*, or *Pax5* genes [10]. Recent studies have also implicated the transcription factor FOXO-1 in early B lymphopoiesis. FOXO-1-deficient mice also show a developmental block at the pro-B cell stage [11]. Moreover, it has been reported that FOXO-1 regulates *Rag1* and *Rag2* expression [44].

Recent evidence indicates that the network of transcription factors Pax5, E2A and EBF also cooperate to regulate their target genes. For example, E2A, EBF, and Pax5 coordinate epigenetic events that lead to the expression of *mb-1*, which encodes the Ig*α* subunit of the pre-BCR and BCR [45]. *mb-1* is methylated at CpG dinucleotides in HSCs and is gradually demethylated during B cell commitment correlating with its pattern of expression [21]. EBF and E2A contribute to the CpG demethylation and nucleosomal remodeling of the *mb-1* promoter, an event necessary for its transcriptional activation by Pax5. ATP-dependent chromatin remodeling complexes have also been implicated in EBF and Pax5-mediated regulation of the *mb-1* gene [21]. Knockdown of Brg1 and Brm, the catalytic subunits of the SWI/SNF chromatin-remodeling complex interfere with EBF and Pax5-mediated activation of *mb-1.* In contrast, knockdown of Mi-2, the catalytic subunit of the Mi-2/NuRD chromatin-remodeling complex, enhances chromatin accessibility and demethylation of the *mb-1* promoter and its transcription in response to both transcription factors [21]. These results are consistent with a model in which the SWI/SNF and Mi-2/NuRD chromatin remodeling complexes play antagonistic regulatory roles to enable or limit the reprogramming of target genes by EBF and Pax5 during B cell development [21]. The B-cell-specific gene *Cd19 *is another example of a gene that is epigenetically regulated during early B cell development. *Cd19* encodes a cell surface protein that participates in signal transduction mechanisms via the BCR and pre-BCR. Chromatin remodeling at the upstream enhancer sequences of *Cd19* occurs in multipotent progenitors [22]. This chromatin remodeling has been shown to facilitate the recruitment of E2A to this locus followed by EBF and Pax5 recruitment [22]. Interestingly, the *Cd19* promoter is transcriptionally activated only after Pax5 binding. In this context, Mercer et al. recently reported that the monomethylation of H3K4 (H3K4me) at the enhancer regions of cell lineage-specifying genes is the main epigenetic mark, which is associated with their specific expression pattern throughout the lymphoid differentiation program [46]. Taken together, these reports provide clear examples of how B cell lineage-specific transcription factors cooperatively mediate the epigenetic regulation of target genes during B lymphopoiesis.

The recent advances in ultrasequencing technologies are helping to draw a global picture of how the networks of transcription factors modify the chromatin of their target genes. The laboratory of Cornelis Murre, using a ChIP-seq experimental approach, has elucidated how the network of transcription factors E2A, EBF and FOXO-1 orchestrates B cell commitment [18]. They found that during the transition of pre-pro-B cell to pro-B cells, E2A-associated genes become monomethylated at lysine 4 on H3 (H3K4me), a mark mainly found on gene enhancer elements. Subsequently, EBF and FOXO1 are involved in the enrichment of active histone modifications such as H3K4me3 on B-cell-specifying genes, such as *Pax5* [18]. Recently, Treiber and colleagues have shed light on the EBF-mediated epigenetic regulation of its target genes [47]. They classified EBF targets as activated, repressed, or primed genes. They observed that, in pro-B and pre-B cells, the “activated” genes are enriched in H3K4me3 and H3 acetylation active marks and show low levels of the repressive mark H3K27me3 [47]. In contrast, the “repressed” genes show the opposite pattern of histone modifications. The “primed” genes are enriched in the gene enhancer mark H3K4me in pre-B and pro-B cells and enriched in H3K4me3 and H3 acetylation in mature B cells [47]. The identification of the epigenetic regulators recruited by transcription factors to mediate gene expression changes during B lymphopoiesis remains to be addressed.

Other epigenetic marks, such as ubiquitination of Histone H2A, have proved to play a role in early B cell development. Jiang et al. pointed out that the histone H2A deubiquitinase MYSM1 is an important factor in B cell development [48]. *Mysm1* knockout mice show a drastic decrease in the number of B cells in the bone marrow, peripheral blood, and lymph nodes [48]. The authors concluded that MYSM1 antagonizes the action of the polycomb repressive complex 1 (PRC1) on the *Ebf1 *promoter, enabling lineage-specific transcription factors, such as E2A, to be recruited to the *Ebf1 *locus and to induce its transcription [48].

Early B cell development is also known to be regulated by microRNAs. Mice deficient in Ago2, which encodes a protein essential for microRNA biogenesis and function, display a block in B cell development at the pro-B cell stage [49]. Consistent with this, specific deletion of Dicer in pro-B cells, which abolishes the entire miRNA network in B cells, results in a complete block of B cell differentiation at the transition from pro-B to pre-B-cells [50]. Another study reported that miR-181, one of the approximately 100 microRNAs known to be expressed in mouse bone marrow cells, is more abundant in the B cell lineage than in other cell types [51]. Transplantation of multipotent hematopoietic progenitors overexpressing miR-181 into lethally irradiated mice resulted in an increase in the number of B cells [51]. Thus, miR-181 appears to target and repress the transcripts of critical genes involved in generating B cells. A similar experimental approach was used to show that another microRNA, miR-150, which is expressed in mature B and T cells, can block B cell differentiation at the pro-B cell stage when expressed prematurely [52]. Accordingly, the laboratory of Klaus Rajewsky reported that miR-150 plays a role during B cell differentiation through its action on *c-Myb* expression [53]. Other miRNAs have been associated with the early development of B cells. For instance, miR-34a ablation results in a developmental block at the pre-B cell stage, and miR-17-92 knockout mice exhibit a block in pro-B cells [54, 55]. They regulate the *Foxbp1* and *Bim *and* PTEN* genes, respectively, which are known to have a role in B cell differentiation [54, 55]. Recently, Kuchen et al. have elucidated the microRNAome during lymphopoiesis at the genome-wide scale, leading to the identification of miRNAs that are primed for expression at different stages of differentiation [56]. They reported that miRNA expression is tightly regulated by epigenetic modifications. In particular, they showed that the repressive mark H3K27me3 is associated with the gene silencing of lineage-inappropriate miRNA during lymphopoiesis [56]. However, they also observed that active epigenetic regulation by the presence of H3K4me also occurs in some of the microRNAs “primed” to be expressed. On the basis of the restrictive expression and abundance of miRNAs during B cell lineage specification, miR-320, miR-191, miR-139 and miR28 appear to be potential regulators of B cell differentiation [56]. The transcripts targeted by key miRNAs for the early differentiation of B cells remains to be identified.

## 4. B Cell Development: Pax5 in the Maintenance of B Cell Identity

The transcription factor Pax5 is essential for maintaining the fate of B cells and is therefore considered to be “the guardian of B cell identity” [57]. Its expression gradually increases in a stepwise manner during B cell development. Pax5 expression is first detected at the early pro-B cell stage and maintained up to the mature B cell stage. Pax5 knockout mice show a block in B cell development at the pro-B stage [12]. Pax5−/− pro-B cells express both E2A and EBF transcription factors, as well as their target genes. In contrast, E2A−/− and EBF−/− derived cells do not express Pax5. Collectively, these data indicate that Pax5 is a target for both transcription, factors. The laboratory of Meinrad Busslinger has shed light on the molecular mechanisms involved in the gradual expression of Pax5 during B cell development. In particular, they have identified an enhancer in the *Pax5* locus, which in combination with the promoter, recapitulates B lymphoid Pax5 expression [58]. Interestingly, the *Pax5* enhancer is silenced by DNA methylation in embryonic stem cells, while it becomes activated in multipotent hematopoietic progenitors. The presence of consensus binding sites for the transcription factors PU.1, IRF4, IRF8, and NF-*k*B within the *Pax5* enhancer suggests that these transcription factors play a role in sequential enhancer activation in hematopoietic progenitors and during B cell development [58]. At the onset of pro-B cell development the transcription factor EBF1 induces chromatin remodeling at the *Pax5* promoter region. In non-B cells, Polycomb group proteins repress the *Pax5* promoter region [58].

In addition to the epigenetic regulation of its expression during B cell development, Pax5 induces the establishment of a B cell-specific transcription program that is associated with the suppression of inappropriate genes of alternative lineages, thereby ensuring its role in maintaining B cell identity and differentiation. Using gene expression microarrays and genome-wide ChIP-on-chip experimental approaches, the laboratories of Busslinger and Nutt have described the complex gene regulatory network regulated by Pax5 during B lymphopoiesis [59–61]. These studies have identified genes that are activated or repressed by Pax5 in wildtype pro-B cells. Pax5-activated genes appear to encode transcription factors and key proteins involved in B cell signaling, adhesion, migration, antigen presentation and germinal-center B cell formation [59, 61]. However, Pax5-repressed genes encode secrete proteins, cell adhesion molecules, signal transducers and nuclear proteins that are specific to erythroid, myeloid, and T cell lineages [59, 61]. Pax5-activated genes in pro-B cells were found to be enriched with epigenetically active marks, including H3K9ac, H3K4me2 and H3K4me3 [60]. Importantly, in Pax5-deficient pro-B cells, these active histone marks were dramatically reduced or lost, indicating that Pax5 is essential for guaranteeing the active chromatin structure at its target genes. These findings demonstrate that Pax5 is a master regulator of B cell identity, which, in conjunction with epigenetic regulators, coordinates a B-cell-specific target gene transcription program. Recently, McManus and colleagues have described the epigenetic mechanisms mediated by Pax5 during B lymphopoiesis [19]. By using a ChIP-on-chip analysis, they have identified Pax5 target genes in committed pro-B cells. The authors also apply a proteomic approach to identify Pax5 interacting partners. They found that Pax5 interacts with the members of the SWI/SNF chromatin remodeling complex Brg1. BAF57 and BAF170. They also reported that PAX5 recruits the NCoR1 repressor complex with its associated HDAC3 activity to repressed its target genes [19]. This study has provided novel important insight into the regulatory network and epigenetic regulation, by which Pax5 directly controls B-cell commitment at the onset of B lymphopoiesis.

The mechanism by which Pax5 mediates transcriptional repression of targets has also been informatively examined using a candidate gene approach. One of the important target genes repressed by Pax5 in B cells is the colony-stimulating factor receptor 1 gene (*csf1r* or *c-fms*), a gene essential for macrophage development. *Csf1r* is expressed at low levels in HSCs and downregulated in all nonmacrophage cell types. In HSCs, MPPs, CMPs, and CLPs the *Csf1r *promoter is bound by transcription factors and its chromatin structure in an active conformation [62]. However, the *Csf1r *gene is silenced during B cell differentiation. Interestingly, an intronic antisense transcription unit that is differentially regulated during lymphopoiesis overlaps with regions of de novo DNA methylation in B cells, highlighting DNA methylation as a mechanism for *Csf1r *silencing during B cell development. Despite being silenced, *Csf1r *chromatin remains in a poised or primed conformation even in mature B cell stages. Importantly, *Csf1r *expression can be reactivated by conditional deletion of the transcription factor Pax5 [62]. Pax5 was shown to bind the *Csf1r *gene directly, resulting in loss of RNA polymerase II recruitment and binding of myeloid transcription factors at cisregulatory elements [63]. Finally, Pax5 in conjunction with linker histone H1 also coordinates DNA methylation and histone modifications in the 3′ regulatory region of the immunoglobulin heavy chain locus and thus epigenetically regulates the IgH locus [64].

## 5. B Cell Development: Terminal Differentiation

The completion of V(D)J recombination and expression of the BCR on the surface of B cells marks the beginning of antigen-dependent B cell development. From this point, B cells undergo terminal differentiation dependent on signals emanating from the BCR after antigen triggering [65]. Peripheral B cells, without antigen-mediated signaling, are in a resting state [66]. Once activated, they either initiate the germinal center (GC) reaction or differentiate into antibody-secreting plasma cells. Entry into the GC reaction is regulated by Bcl6, whereas the generation of antibody-secreting plasma cells is controlled by Blimp-1. Bcl6 and Blimp-1 both act as transcriptional repressors and work in a mutually exclusive manner [67, 68].

After antigen triggering, Bcl6 is upregulated in some B cells that then enter the GC reaction [13–15]. In contrast, cells in which Bcl6 is not upregulated undergo differentiation into plasma cells [69, 70]. From a mechanistic angle, Bcl6 has been shown to interact with the chromatin remodeling complex Mi-2/NuRD in GC B cells, leading to the repression of specific genes that are characteristic of plasma cells [71, 72]. This Mi-2/NURD-mediated repression requires the recruitment of histone deacetylases HDAC1 and HDAC2 [71, 72].

After activation of GC B cells Bcl-6 expression is downregulated in association with the expression of its target gene Blimp-1. Once expressed, Blimp-1 represses the gene expression program of mature B cells, thereby promoting plasma cell differentiation [16, 17]. Mechanistically, Blimp-1 exerts its repressive transcriptional activity by recruiting regulators and coordinating epigenetic modifications at its target genes. PRD1-BF1, the human orthologue of Blimp-1, silences the interferon beta gene in response to viral infection by recruiting the histone methyltransferase (HMTase) G9a to the interferon-beta promoter, resulting in H3K9me [73]. Blimp-1 has also been found in a complex with the arginine histone methyl transferase Prmt5, although the functional significance of this interaction in B cells is not clear [74]. The histone lysine demethylase LSD1 has also been shown to interact with Blimp-1 [75]. Chromatin immunoprecipitation (ChIP) experiments indicated that Blimp-1 and LSD1 share some target genes leading to a more accessible chromatin structure [75]. Importantly, disruption of the Blimp-1-LSD1 interaction resulted in attenuated antibody secretion of the cells, highlighting the functional relevance of this interaction for B cell function.

In the last few years, additional transcription factors have emerged as being involved in B cell terminal differentiation. It has been reported that IRF4 and XBp1 control the maintenance of plasma cell identity. IRF4 is responsible for BLIMP-1 induction and, in conjunction with XBp1, determines the fate of the plasma cell. The network of transcription factors Pax5, Bach2, and Bcl6 direct B cell development into germinal center cells. It has been shown that Pax5 induces Bach2 expression after B cell activation, which in turn cooperates with Blc6 to repress Blimp-1 expression promoting activation-induced cytidine deaminase (AID) expression and antibody class switch [76, 77].

MicroRNAs are also involved in the terminal differentiation of B cells. Peripheral B cells in transit to their final maturation can give rise to two functionally distinct peripheral populations: follicular (FO) or marginal zone (MZ) B cells. FO versus MZ fate decision is functionally coupled to BCR signaling and it has been suggested that B cells bearing BCRs with autoreactive specificities are preferentially driven into a MZ fate [78]. In 2010, Belver and colleagues generated conditional Dicer-deficient mice at later stages of B cell development [79]. They observed that miRNA metabolism is important for such developmental stage since these mice presented an impairment in the generation of follicular B cells and an overrepresentation of marginal zone B cells. Accordingly, another phenotypic feature of these mice was the presence of high titers of autoreactive antibodies [79]. They identified miR185 as an important factor for the correct BCR-mediated development of B cells.

## 6. B Cell Reprogramming and Transdifferentiation

**Figure 2:**
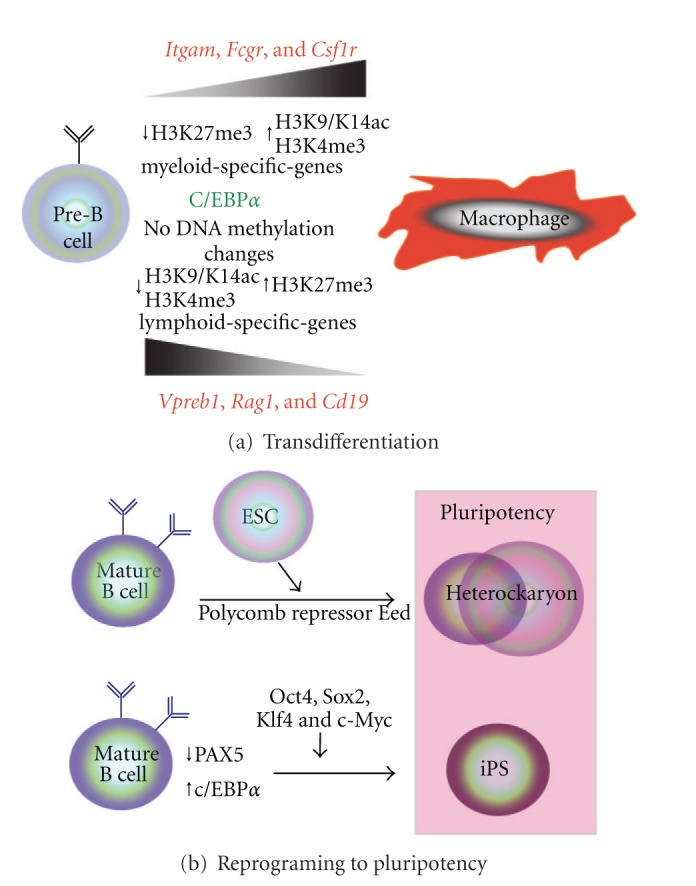
Transdifferentiation and reprogramming of B cells. (a) Ectopic expression of C/EBP in pre-B cells induces their transdifferentiation into macrophages. Epigenetic changes during the process are shown. (b) B cells can be reprogrammed to pluripotency by fusion with ESCs (heterokaryon) or by transgenic induction of Oct4, Sox2, Klf4 and c-Myc (iPS).

Since 1987, when the possibility of reprogramming specialized cells by the expression of a linage-specific transcription factor was first reported, many studies have tried to understand the molecular mechanisms that control all the processes involved. Due to the high developmental complexity that characterizes the hematopoietic system, it constitutes a model system with which study cell reprogramming and transdifferentiation in greater depth. In 1995, it was reported that overexpression of the erythroid lineage-specific transcription factor GATA-1 in myeloid leukemia cells induced their reprogramming into the megakaryocytic/erythroid lineage [24]. Subsequently, Nutt et al. reported that Pax5-defective pro-B cells differentiated into functional macrophages, granulocytes, natural killer cells, osteoclasts, and dendritic cells when specific cytokines were added to the culture medium [26]. Some years later, using knock-in and lineage-tracing technologies in mice, Xie and colleagues were able to demonstrate *in vivo* reprogramming of intrasplenic mature B cells into macrophages by the overexpression of the myeloid transcription factor C/EBP*α* [80]. More recently, the same laboratory generated a robust reprogramming system in which murine pre-B cells were converted into functional macrophages by the overexpression of C/EBP*α* [25] (Figure 2). This cellular conversion has been considered a transdifferentiation event since it is irreversible and does not require the retrodifferentiation of pre-B cells to previous progenitor stages [81]. Using the cellular system generated in Graf's laboratory, Radríguez-Ubreva and colleagues performed a high-throughput methylation analysis to study changes in DNA methylation during the transdifferentiation of pre-B cells into macrophages [82]. Surprisingly, they did not find any significant changes in DNA methylation during cellular conversion. However, they were able to identify the expected histone modifications in the genes that had previously been described to be upregulated or downregulated during the process. In particular, they reported an increase in the enrichment of the active histone marks H3K9/K14ac and H3K4me3, at the promoters of upregulated macrophage-specific genes, whereas a reduction of these modifications was observed in the B-cell-specific downregulated genes. In contrast, the repressive mark H3K27me3 was found to be enriched in the B cell downregulated genes and reduced in the upregulated macrophage-specific genes [82] (Figure 2). This study suggests that histone regulators are able to overcome the repressive effect of DNA methylation in macrophage-specific genes in the converted cells. It also establishes an important difference from the process of reprogramming towards pluripotency in which promoter DNA demethylation plays a crucial role.

In this regard, Hanna and colleagues demonstrated that pro-B and pre-B cells can be reprogrammed into induced pluripotent stem (iPS) cells by the expression of the transcription factors Oct4, Sox2, Klf4, and c-Myc [27]. Interestingly, the expression of the four factors in mature B cells does not result in the reprogramming of mature B cells to pluripotency. They found that expression of c/EBP*α* in conjunction with the four “reprogramming” factors is necessary to generate iPS cells [27] (Figure 2). iPS cell lines derived from immature and mature B cells show promoter demethylation of the stem cell markers Oct4 and Nanog, whereas both promoters are heavily methylated in the original B cells. Finally, in mature B cells the promoter region of *Pax5 *shows high and low levels of enrichment for the active mark H3K4me3 and the repressive mark H3K27me3, respectively. Conversely, equivalent enrichment of both histone modifications was observed in iPS lines derived from mature B cells [27]. This study raises the challenging question of how B lymphocytes at different developmental stages differ in their epigenetic landscape and how one factor can overcome this divergence to allow reprogramming into pluripotent cells.

A number of studies using experimental heterokaryons, in which a somatic cell is reprogrammed towards pluripotency by fusion with mouse embryonic stem (ES) cells, have also been used to reprogram B lymphocytes into pluripotent cells. The laboratory of Amanda Fisher has shown that when mouse ES cells are fused with human B lymphocytes the expression of human pluripotent-associated genes is rapidly induced [83]. Recently, the same group has elucidated some of the epigenetic mechanisms underlying this reprogramming process. They showed that deletion of Eed, Suz12, Ezh2, and Ring1A/B, which are members of either the polycomb repressor complex PRC1 or PRC2, in mouse ES cells abolishes their capacity to induce human B lymphocyte reprogramming towards pluripotency [84] (Figure 2).

## 7. Concluding Remarks

The impressive advances in genome-wide methods and the latest generation of ultrasequencing techniques are opening up new, and challenging lines of research focused on the elucidation of the epigenetic mechanisms underlying B cell differentiation and reprogramming. Many questions remain to be answered. Is there a specific “epigenetic signature” for the different cellular states comprising B cell development? How can lymphoid-specific transcription factors orchestrate the epigenetic machinery at different genes and genome regions to facilitate the choice to differentiate into a particular cellular lineage? Is the expression of epigenetic regulators lineage-specific? Epigenetic modification analyses, genome-wide RNA and ChIP-Seq studies, quantitative proteomics, and systematic functional studies offer us the opportunity to obtain high-quality measurements that will provide us with a draft of the “epigenetic-transcriptional” program that controls B cell development and reprogramming. Finally, conditional gene inactivation in mice will reveal the role of specific epigenetic regulators during B cell development. Thus, new regulatory networks connecting epigenetic and transcription factors seem likely to be revealed in the context of B lymphopoiesis in the future.
